# Origin of rice (*Oryza sativa* L.) domestication genes

**DOI:** 10.1007/s10722-017-0518-0

**Published:** 2017-05-17

**Authors:** Peter Civáň, Terence A. Brown

**Affiliations:** Manchester Institute of Biotechnology, School of Earth and Environmental Sciences, University of Manchester, Manchester, UK

**Keywords:** Cultivated rice, Domestication genes, Origins of cultivation, *Oryza sativa*, Whole-genome sequence data, Wild rice

## Abstract

A number of genes that contribute to the domestication traits of cultivated rice have been identified. These include *Sh4*, *Rc*, *PROG1* and *LABA1*, which are associated with non-shattering rachis, white pericarp, erect growth and barbless awns, respectively. The mutations giving rise to the “domestication alleles” of these genes are either invariable in cultivated rice, or have variability that is strictly associated with the phenotypic trait. This observation forms the basis to those current rice domestication models that envisage a single origin for the domesticated phenotype. Such models assume that the domestication alleles are absent or rare in wild rice, emerged under cultivation and spread across all rice groups by introgressive hybridization. We examined whole-genome sequencing datasets for wild and cultivated rice to test the former two assumptions. We found that the *rc* and *laba1* alleles occur in wild rice with broad geographical distribution, and reach frequencies as high as 13 and 15%, respectively. These results are in agreement with previous observations of the *prog1* and *sh4* domestication alleles in wild populations. We also show that the diversity of the genomic regions surrounding the *rc*, *laba1*, *prog1* and *sh4* alleles in wild accessions is greater than that in cultivated rice, suggesting that these alleles emerged prior to domestication. Our findings indicate that the possibility that independent rice groups obtained identical domestication alleles directly from the wild population needs to be considered.

**Electronic supplementary material:**

The online version of this article (doi:10.1007/s10722-017-0518-0) contains supplementary material, which is available to authorized users.

## Introduction

Cultivated Asian rice (*Oryza sativa* L.) can be divided into several groups based on different culinary properties, ecology and genetics—*O. sativa* subsp. *indica* Kato; subsp. *japonica* Kato (with *tropical* and *temperate* subgroups); *aromatic* rice with specific flavours popular in India and Pakistan (basmati) and Iran (sadri), usually treated as a subgroup of subsp. *japonica*; and *aus* rice consisting of early-maturing, draught-tolerant ecotypes previously considered to be a subgroup of subsp. *indica* (Garris et al. 2005; Zhao et al. 2011; Civáň et al. 2015). Current ideas regarding the domestication of *O. sativa* are strongly driven by analyses of so called “domestication genes”, i.e. genes contributing to the domestication phenotype of cultivated rice. The domestication phenotype is a set of characteristics differentiating cultivated rice from its wild progenitor *O. rufipogon* Griff., resulting from selective pressures imposed by humans during domestication. The most prominent of these changes are growth habit, reduction of seed dormancy, overall yield, quality of rachis, hulls and awns, adaptations to diverse habitats, and the culinary properties of the grain. Although most of these traits are quantitative in nature and hence controlled by a network of genes interacting with the environment, several major genes determining alternative modes of phenotypic variability in cultivated rice have been identified. Well-characterised examples of such major genes are *Sh4* (Li et al. 2006), *Rc* (Sweeney et al. 2006), *PROG1* (Tan et al. 2008) and *LABA1* (Hua et al. 2015), whose recessive alleles are associated with non-shattering rachis, white pericarp, erect growth and barbless awns, respectively. Functional polymorphisms associated with the phenotypic changes have been identified as either microdeletions disrupting the open reading frame of the coding sequence (*rc*, *laba1*), a mutation causing an amino acid change in the protein (*sh4*), or a set of candidate mutations in the coding sequence and promoter region (*prog1*). These mutations are either invariable in cultivated rice (*sh4*, *prog1*), or their variability is strictly associated with the phenotypic trait (*rc*, *laba1*). For example, all cultivated rice with a white pericarp (with exceptions in *aus*) has a 14 bp deletion in the *Rc* coding sequence, while all cultivated rice with a red pericarp has the reading frame uninterrupted by the deletion (Sweeney et al. 2007). Since the red grain colour is ubiquitous in *O. rufipogon*, Sweeney et al. (2007) did not search for the 14 bp deletion in wild populations and assumed that the *rc* allele emerged under cultivation. Based on this assumption, they compared the *Rc* haplotypes found in red *japonica* and red *indica* cultivars and concluded that the *rc* variant emerged in subsp. *japonica* and was transferred to subsp. *indica* and partially also to *aus* by introgressive hybridization (Sweeney et al. 2007). Similarly, Oikawa et al. (2015) assumed that the allelic variant of the *Kala4* gene responsible for black pigmentation in some rice cultivars emerged during or after domestication, and did not examine genotypes in wild populations (where black pigmentation has not been found).

Since it has been shown that gene variants beneficial for cultivation can be selected from the standing variation of the progenitor (Weber et al. 2007, 2008; Studer et al. 2011) and that the genotype-phenotype association in wild populations may be obscured by interacting genes (Zhu et al. 2012), the assumption that any domestication allele emerged under cultivation is rather risky. Examination of allelic variability in wild populations has been conducted for other rice genes, although with various sample sizes. Hua et al. (2015) examined 43 *O. rufipogon* accessions and did not find a single case with the *laba1* deletion. However, Tan et al. (2008) sequenced the *PROG1* coding sequence in 30 accessions of wild rice with prostrate growth and found 14 cases where the wild haplotypes are identical or similar to the uniform allele of cultivated rice. This led the authors to conclude that other gene(s) may be involved in control of prostrate growth in wild rice. A similar conclusion has been reached regarding the *Sh4* gene since multiple other loci affecting the degree of seed shattering have been discovered (Konishi et al. 2006; Subudhi et al. 2014; Yao et al. 2015) and it has been shown that the “non-shattering” *sh4* allele is relatively common in wild populations, being present in 44 of 166 wild accessions tested by Zhu et al. (2012).

Knowing if the recessive variants of the *Sh4*, *PROG1*, *Rc* and *LABA1* genes naturally occur in wild populations is crucial for interpretation of the rice domestication process. Absence of these alleles in wild populations would indicate that the mutations indeed emerged during domestication. Allelic uniformity in cultivated rice would then imply either a single origin of *O. sativa*, or spread of the domestication alleles among different groups of *O. sativa* by introgressive hybridization, as has been suggested (Sweeney et al. 2007; Fujino et al. 2010; Hua et al. 2015; Oikawa et al. 2015; Si et al. 2016). However, if the association between the mutations and the phenotype is not strict in *O. rufipogon*, and the “domestication alleles” are found in wild populations—as is the case with *sh4* and *prog1*—then it is more difficult to make conclusions about the origin of those alleles in different cultivated groups. For example, subsp. *indica* could have received a domestication allele from subsp. *japonica* by introgressive hybridization (or vice versa), or both groups could have outsourced the allele from their wild progenitors in separate domestication events. Despite the fact that the *prog1* and *sh4* alleles are found in *O. rufipogon* and the occurrence of other domestication alleles in wild rice has not been thoroughly checked, models proposing a single origin of cultivated rice followed by spread of the domestication alleles by introgressive hybridization have become widely accepted (Kovach et al. 2007; Sang and Ge 2007; Izawa 2008; Huang et al. 2012; Gross and Zhao 2014). Here we examine the occurrence of the *sh4*, *prog1*, *rc* and *laba1* alleles in *O. rufipogon* by searching published whole-genome sequencing datasets, and based on our findings suggest that the possibility that independent rice groups obtained identical domestication alleles directly from the wild population needs to be considered.

## Methods

We downloaded raw sequence data for 1543 wild and cultivated rice accessions (Huang et al. 2012) from the Sequence Read Archive (ERP001143, ERP000729, ERP000106) and converted each file into FASTQ format using the fastq-dump command in sratoolkit 2.3.5. The full FASTQ dataset consisted of 9.174 billion Illumina reads totalling 2.129 TB and was processed on a Linux platform. Low-quality regions were removed by Trimmomatic-0.33 (Bolger et al. 2014).

These data sets have low sequencing depth (mean 1× and 2× for *O. sativa* and *O. rufipogon*, respectively; Huang et al. 2012) which does not allow reconstruction of entire genes or ungapped haplotypes. Nonetheless, known single nucleotide polymorphisms (SNPs) or indel variants can be scored in a subset of the samples where sequencing reads are available for the particular locus. Given the sample size (460 accessions of wild rice; 519 subsp. *indica*; 482 subsp. *japonica*; 30 *aus*; 5 *aromatic*; 47 other/unassigned), estimates of allelic frequencies can be obtained for subsp. *indica*, subsp. *japonica* and wild rice, despite the prevalence of missing data.

To determine indel variants of the *Rc* gene, we searched each of the 1543 trimmed FASTQ datasets using the *grep* command with 30 bp words (perfect match) spanning the 14 bp indel site in the *Rc* gene (5′-AAAGGCGCAAGTGGATGCCATCCAAGGTGA-3′ and reverse complement, matching the variant with deletion; 5′-CAAGTGGAACGCGAAAAGTCGGTGCCATCC-3′ and reverse complement, matching the wild type). Identified matches were further verified by aligning reads to reference sequences of the *Rc* gene with and without the deletion. We also searched for reads perfectly matching a 30 bp sequence spanning the 1 bp indel site in the *LABA1* coding sequence (5′-AGCCATGGCTCTACTCAGTCTCGGTTCAGG-3′ and reverse complement, matching the variant with deletion; 5′-AGCCATGGCTCTACTCCAGTCTCGGTTCAG-3′ and reverse complement, matching the wild type), and verified these matches against the reference sequences for the *LABA1* gene.

To obtain diversity estimates for the *rc, laba1*, *sh4* and *prog1* haplotypes, we searched for variability in 10 kb windows surrounding the causative mutations utilising the SNP matrix published by Huang et al. (2012) (downloaded from the Rice Haplotype Map Project database). The diversity was calculated as the average number of polymorphic sites in a 10 kb window surrounding the causative mutation, weighted per examined accession and the proportion of non-missing data. A maximum-parsimony tree of the *Sh4* locus was constructed from the data published by Zhu et al. (2012) using dnapars program in the PHYLIP package with default parameters (Felsenstein 2005). Only the parsimony-informative sites were used and >1 bp gaps were recoded as single events. A majority consensus tree was constructed from 5171 equally parsimonious trees.

## Results and discussion

We found one or more reads exactly matching the causative indel position in the *Rc* gene for 255 accessions of wild rice (Table 1; Table S1). In 33 cases (12.9%), the *rc* variant with the deletion was detected. Although we do not posses detailed phenotypic information about the wild accessions, we assume that all of them have pigmented pericarp. We therefore show that the *rc* allele does exist in wild rice in moderate frequency and is not necessarily associated with white pericarp, similar to the situation previously reported for the *sh4* and *prog1* alleles. Consequently, the conclusion of Sweeney et al. (2007) that the *rc* allele originated in subsp. *japonica* and later spread to subsp. *indica* by introgressive hybridization is questioned, and the possibility that all cultivated rice groups obtained the *rc* allele directly from their wild progenitors has to be considered.

**Table 1:** Summary of the *Rc* and *LABA1* alleles detected in cultivated groups and wild populations

	*Rc*	*rc* (14 bp deletion)	*rc* diversity	*LABA1*	*laba1* (1 bp deletion)	*laba1* diversity
subsp. *japonica (tropical)*	7 (31.8%)	15 (68.2%)	0.42 (*O. sativa*)	0	24 (100%)	0.44 (*O. sativa*)
subsp. *japonica (temperate)*	15 (11.6%)	114 (88.4%)		50 (58.1%)	36 (41.9%)	
*indica* ^a^	65 (28.5%)	167 (73.2%)		3 (2.3%)	130 (97.7%)	
*aus*	7 (100%)	0		8 (66.7%)	4 (33.3%)	
*aromatic*	0	2 (100%)		0	3 (100%)	
*o. rufipogon* ^b^	218 (87.2%)	33 (13.2%)	1.69	185 (86.0%)	33 (15.3%)	2.07

^a^Four subsp. *indica* accessions were found with both *Rc* alleles (heterozygotes)

^b^One *O. rufipogon* accession was found with both *Rc* alleles, while three accessions had matches with both *LABA1* alleles

In the case of the *LABA1* gene, we found perfect matches for the indel position in 215 wild accessions. In 33 of those (~15%), the variant with the deletion was detected. In contrast, Hua et al. (2015) did not find a single case of the *laba1* allele in their smaller sample of 43 *O. rufipogon* accessions.

**Fig. 1:**
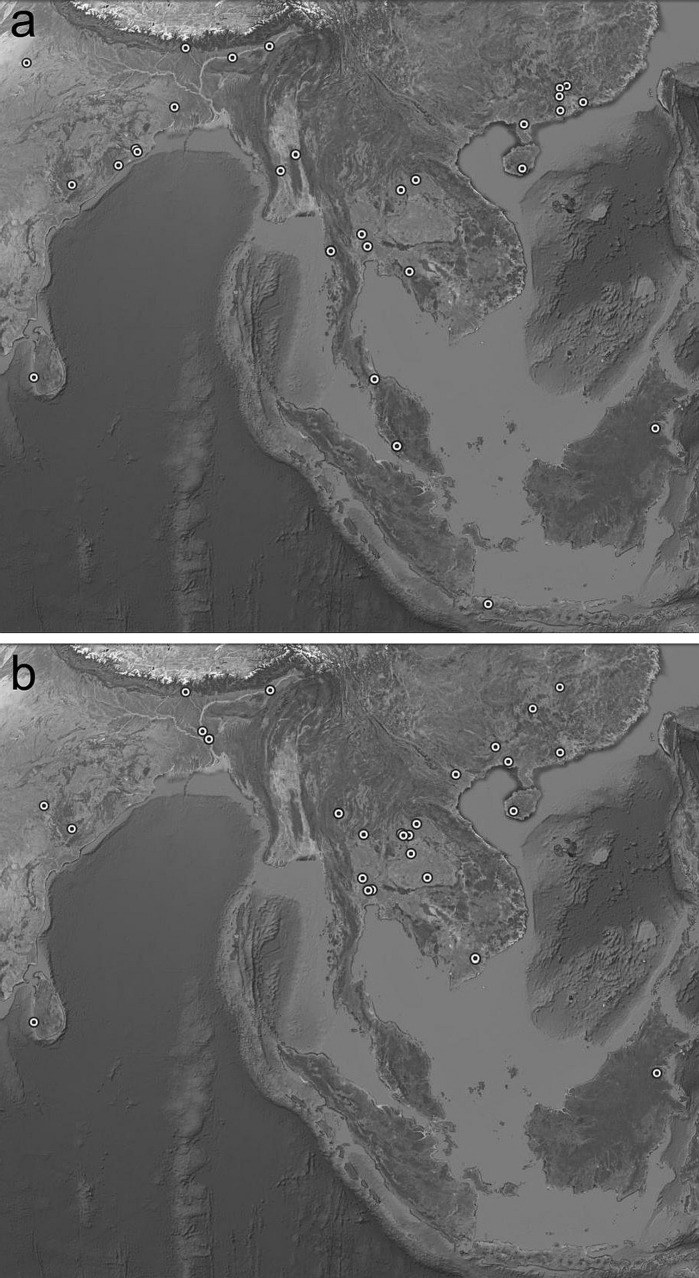
Geographic location of *O. rufipogon* accessions with the *rc* (**a**) and *laba1* (**b**) alleles. In both cases, two Chinese samples are not shown due to unavailable coordinates. Map prepared in Google Earth v7.1.5.1557

Plots of the geographical locations of the wild accessions carrying the *rc* and *laba1* alleles show that both alleles have a relatively broad distribution (Fig. 1). This is consistent with the results of Liu et al. (2015), who found no correlation between genetic groups and geographic regions in wild rice, and ascribed the absence of a phylogeographic pattern to repeated extinctions and re-colonizations of wild populations during Quaternary glacial-interglacial cycles. If this explanation is correct, it may imply that both mutations emerged prior to the last glaciation.

It could be argued that the observation of the “domestication” alleles in wild populations does not necessarily reject the hypothesis of their origin under cultivation. Each of the recessive alleles could have emerged during the domestication process and escaped into wild populations by gene flow. This possibility needs to be evaluated critically. Gene flow from *O. sativa* to its wild relatives has been well documented due to concerns of transgene escape from genetically modified rice (Song et al. 2003; Chen et al. 2004; Wang et al. 2006; Shivrain et al. 2007). The recessive alleles of the *Sh4*, *PROG1*, *Rc* and *LABA1* genes can be transferred by gene flow just like any other genomic segment. However, these alleles are either neutral (if no phenotypic change is manifested) or disadvantageous in wild populations. In the absence of positive selection, it is difficult to conceive that gene flow and retention of the alleles in the wild would occur to the extent resulting in the frequencies reported for *sh4* (~26%; Zhu et al. 2012), *rc* and *laba1* (~13 and 15%, respectively; this study). Moreover, high fixation index (F_ST_) values indicate that the reproductive barrier between cultivated rice and *O. rufipogon* is relatively strong. For example, Huang et al. (2012) calculated that the F_ST_ between subsp. *japonica* and its assumed progenitor population is 0.36, which means that *japonica* rice and its wild progenitor—although sympatric—share less genetic variability than East-Asians do with people from sub-Saharan Africa (F_ST_ = 0.19; Nelis et al. 2009).

**Fig. 2:**
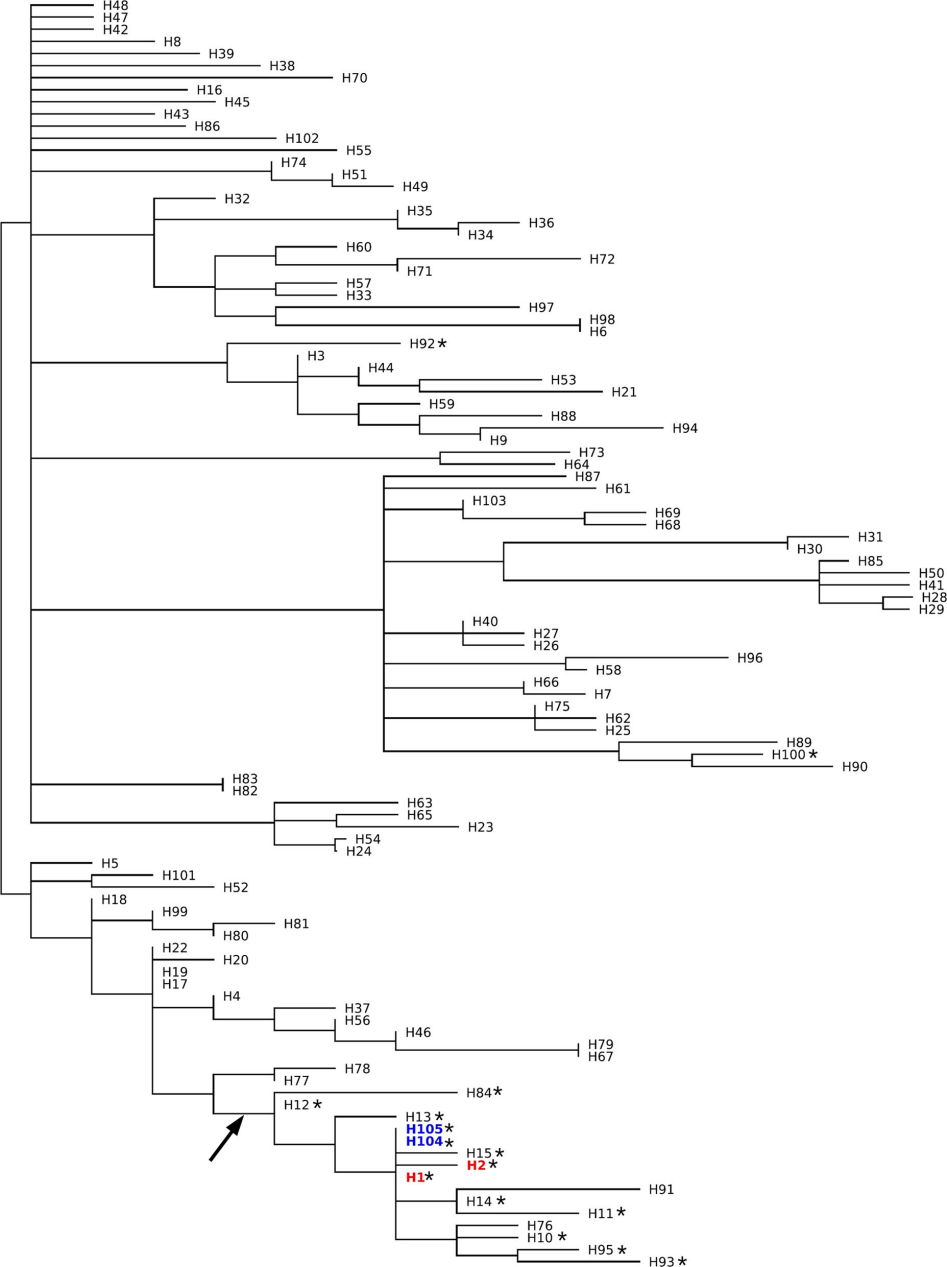
Maximum-parsimony tree constructed from the *sh4* haplotype data published by Zhu et al. (2012). Haplotypes with the asterisk carry T at the FNP (Functional Nucleotide Polymorphism) site identified by Li et al. (2006) (chromosome 4; IRGSP4 position 34,631,527). The haplotypes H104 and H105 (*blue*) are only found in weedy rice. All cultivated rice carries either of the haplotypes H1 and H2 (*red*) that are also found in wild rice. The haplotypes H10, H11, H12, H13, H14, H15, H84, H93 and H95 are only found in wild rice. The inferred origin of the G → T FNP is indicated by an arrow. Two unrelated wild haplotypes also carry T at the FNP site (H92, H100), probably as a result of homoplasy. The haplotypes H76 and H91 probably encountered reverse T → G mutations

The above considerations suggest that the presence of domestication alleles in wild rice is not wholly explained by gene flow from *O. sativa*, but empirical data are needed to confirm this point. One way of addressing the question is by exploration of associated diversity. If the domestication alleles found in *O. rufipogon* are derived from cultivated rice, then their nucleotide diversity in wild populations would not be expected to exceed their diversity in *O. sativa*. On the other hand, if the emergence of the causative mutations pre-dates domestication, then the domestication alleles found in *O. rufipogon* would have higher diversity compared to *O. sativa*. Our estimates of nucleotide diversity indicate that the latter interpretation is correct. The 10 kb regions surrounding the causative deletion contain ~4× more polymorphisms in *rc*-type and *laba1*-type *O. rufipogon* than they do in *O. sativa* (Table 1). For the *sh4* allele, it can be directly demonstrated that the causative G → T substitution changing asparagine for lysine in the protein product emerged in wild rice prior to domestication. This is established by a gene tree constructed from the polymorphism data in the *Sh4* exon, partial intron and ~1.5 kb flanking region published by Zhu et al. (2012). The phylogenetic tree shows that most haplotypes with the G → T substitution form a clade where the sequences found in *O. rufipogon* occupy both the basal and sister positions in respect to the haplotypes found in *O. sativa* (Fig. 2). Furthermore, in the SNP matrix by Huang et al. (2012) we found a polymorphic site approximately 1 kb downstream of the *Sh4* coding sequence (chromosome 4; IRGSP4 position 34,628,688). All 99 subsp. *indica* accessions with data have A at this position, while 46 out of 67 non-missing *japonica* data points are C (68.7%). Both variants are found in *O. rufipogon* (Table S1), indicating that the *sh4* haplotypes of *O. sativa* do not originate from a single wild genotype.

For the *prog1* allele, the causative mutation has not been unambiguously identified from the set of candidate polymorphisms (Tan et al. 2008), and we therefore could not sort the wild population into *prog1/PROG1* classes. Nonetheless, we identified variability that indicates independent genealogical histories in different groups of *O. sativa*. A SNP located 230 bp upstream from the *PROG1* start codon (chromosome 7; IRGSP4 position 2,872,361) is uniform in subsp. *indica* (all 127 accessions with data have C; Table S1) but variable in subsp. *japonica* (58.9% C and 41.1% A; 129 accessions with data). A short distance further upstream—780 bp away from the *PROG1* start codon (chromosome 7; IRGSP4 position 2,872,911)—another polymorphic position was found, this time monomorphic in subsp. *japonica* (all 128 accessions with data have C) and polymorphic in subsp. *indica* (77.6% C and 22.4% T; 143 accessions with data). Both of these positions are variable in *O. rufipogon*. Thus, we observe two haplotypes in subsp. *indica* (TC-*prog1* and CC-*prog1*) and two haplotypes in subsp. *japonica* (CC-*prog1* and CA-*prog1*). Subsp. *indica* could not have obtained the TC-*prog1* haplotype from subsp. *japonica* since it does not occur there, and similarly, subsp. *japonica* could not have obtained the CA-*prog1* haplotype from subsp. *indica*. However, both groups could have obtained both haplotypes from wild rice, which is the most parsimonious explanation.

The diversity associated with *prog1* and *sh4* therefore indicates different genealogical histories for these alleles in subsp. *indica* and subsp. *japonica*, but unfortunately, the whole-genome data sets do not allow conclusions to be drawn about the *aus* group. Interestingly, Sweeney et al. (2007) reported a second mutation disrupting the open reading frame of the *Rc* gene and leading to white pericarp in some *aus* varieties. Similarly, Hua et al. (2015) found several *aus* varieties with the *LABA1* (wild type) allele but barbless awns. These observations indicate that alternative mutations underlie the domesticated phenotype of some *aus* cultivars, in agreement with our previous suggestion that this group has a unique domestication history (Civáň et al. 2015).

In conclusion, we show that the *rc*, *laba1*, *prog1* and *sh4* alleles are moderately frequent in *O. rufipogon* populations, where they display higher associated diversity than that present in *O. sativa*. This evidence suggests that the causative mutations determining white pericarp, barbless awns, erect growth and non-shattering ear in *O. sativa* each emerged in wild rice prior to domestication. Since these mutations are not associated with the domesticated phenotype in wild rice, a group of interacting genes is probably responsible for the phenotypic trait in each case. The implication is that, for each of these traits, selection in cultivated rice acts on a network of alleles rather than at an individual locus. A single gene can still determine the alternative phenotypes, but only in the appropriate allelic background.

Our results broaden the possible scope for models describing the events giving rise to the different groups of cultivated rice. The uniformity of the domestication alleles in cultivated rice has previously constrained those models, with focus largely on schemes in which the domestication phenotype originated in one type of rice and was subsequently transferred to other groups by introgressive hybridization (e.g. Sweeney et al. 2007; He et al. 2011; Huang et al. 2012; Yang et al. 2012; Hua et al. 2015; Oikawa et al. 2015). The underlying assumption on which these models are based, that the different groups of rice could not have acquired domestication alleles from standing variation in the wild population, is clearly incorrect. Conversely, models that propose independent domestications giving rise to subsp. *indica*, subsp. *japonica* and/or *aus* (e.g. Civáň et al. 2015) are not invalidated by the uniformity of the domestication alleles in these different groups.

## Electronic supplementary material

Below is the link to the electronic supplementary material.

Supplementary material 1 (XLSX 93 kb)
